# Clinical study: the impact of goal-directed fluid therapy on volume management during enhanced recovery after surgery in gastrointestinal procedures

**DOI:** 10.3389/abp.2024.12377

**Published:** 2024-03-19

**Authors:** Ming Gao, Minggan Chen, Gang Dai, Dengfeng Zhu, Yiting Cai

**Affiliations:** Department of Gastrointestinal Surgery, Chongming Hospital Affiliated to Shanghai University of Medicine and Health Sciences, Shanghai, China

**Keywords:** accelerated rehabilitation surgery, goal-directed fluid therapy, vstroke volume variation, cardiac index, pneumoperitoneum, fluid supplement volume, vasoactive drug

## Abstract

**Background:** Goal-directed fluid therapy, as a crucial component of accelerated rehabilitation after surgery, plays a significant role in expediting postoperative recovery and enhancing the prognosis of major surgical procedures.

**Methods:** In line with this, the present study aimed to investigate the impact of target-oriented fluid therapy on volume management during ERAS protocols specifically for gastrointestinal surgery. Patients undergoing gastrointestinal surgery at our hospital between October 2019 and May 2021 were selected as the sample population for this research.

**Results:** 41 cases of gastrointestinal surgery patients were collected from our hospital over 3 recent years. Compared with T1, MAP levels were significantly increased from T2 to T5; cardiac output (CO) was significantly decreased from T2 to T3, and significantly increased from T4 to T5; and SV level was significantly increased from T3 to T5. Compared with T2, HR and cardiac index (CI) were significantly elevated at T1 and at T3–T5. Compared with T3, SVV was significantly decreased at T1, T2, T4, and T5; CO and stroke volume (SV) levels were increased significantly at T4 and T5. In this study, pressor drugs were taken for 23 days, PACU residence time was 40.22 ± 12.79 min, time to get out of bed was 12.41 ± 3.97 h, exhaust and defecation time was 18.11 ± 7.52 h, and length of postoperative hospital stay was 4.47 ± 1.98 days. The average HAMA score was 9.11 ± 2.37, CRP levels were 10.54 ± 3.38 mg/L, adrenaline levels were 132.87 ± 8.97 ng/L, and cortisol levels were 119.72 ± 4.08 ng/L. Prealbumin levels were 141.98 ± 10.99 mg/L at 3 d after surgery, and 164.17 ± 15.84 mg/L on the day of discharge. Lymphocyte count was 1.22 ± 0.18 (10^9^/L) at 3 d after surgery, and 1.47 ± 0.17 (10^9^/L) on the day of discharge. Serum albumin levels were 30.51 ± 2.28 (g/L) at 3 d after surgery, and 33.52 ± 2.07 (g/L) on the day of discharge.

**Conclusion:** Goal-directed fluid therapy (GDFT) under the concept of Enhanced Recovery After Surgery (ERAS) is helpful in volume management during radical resection of colorectal tumors, with good postoperative recovery. Attention should be paid to the influence of pneumoperitoneum and intraoperative posture on GDFT parameters.

## Introduction

During gastrointestinal surgery, patients are influenced by various factors, such as their psychological state, anesthesia, surgical procedures, and pain. As a result, they may experience different levels of illness-related effects. These effects manifest in the form of a robust surgical stress response, leading to the production of significant amounts of stress hormones in patients' bodies. Consequently, symptoms such as elevated blood pressure, inadequate oxygen supply, and accelerated heart rate may arise, ultimately impacting the surgical outcomes (Pu et al., 2020; Daca-Alvarez et al., 2022; Ding et al., 2022; Erdem et al., 2022). Therefore, it is particularly important to implement effective perioperative management for patients undergoing gastrointestinal surgery (Ayala et al., 2022).

Fluid therapy plays a crucial role in ensuring the safety of patients during surgery and facilitating postoperative recovery within the context of Enhanced Recovery After Surgery (ERAS) protocols (Ashok et al., 2020). Consequently, this factor has garnered significant attention not only from surgeons but also from anesthesiologists (Feldheiser et al., 2016). In recent years, with the widespread adoption of the ERAS concept, there has been a surge in the number of medium-to high-risk patients requiring surgical interventions, as well as an increase in the complexity and volume of surgeries performed. This trend has led to a growing interest among scholars in exploring and researching various aspects of fluid therapy (Gustafsson et al., 2019). Building upon traditional fluid replacement and restrictive fluid therapy approaches, the concept of goal-directed fluid therapy (GDFT), which offers more precise and accurate administration, has gained widespread recognition (Arena et al., 2021).

GDFT, or goal-directed fluid therapy, refers to an individualized rehydration approach that relies on monitoring hemodynamic indicators to assess the body’s fluid requirements (Di et al., 2020). The effectiveness of GDFT within the ERAS framework often hinges on various common risk factors associated with both the surgery and the patient (Giusto et al., 2021). Previous research has demonstrated that personalized GDFT during surgical procedures can yield significant benefits for patients categorized as medium-to-high risk (Bisgaard et al., 2020; Jiang et al., 2021). Consequently, recent studies have primarily focused on investigating the application of GDFT in ERAS protocols for major surgery involving middle- and high-risk patients (Aaen et al., 2021). This present study aimed to examine the impact of goal-directed fluid therapy on volume management during ERAS in gastrointestinal surgery.

## Materials and methods

### General information

This study was approved by the Medical Ethics Committee of our hospital (NO: 2020051617), and all patients signed an informed consent form. Adult patients who were scheduled to undergo surgery in Gastrointestinal Procedures under general anesthesia from October 2019 to May 2021 were selected, regardless of gender and ASA score (class I or II).

### Exclusion criteria

Severe uncontrolled cardiovascular, endocrine, or respiratory disease; severe digestive tract obstruction; significant liver and kidney dysfunction; abnormal blood coagulation function; contraindications for artery puncture and central vein catheterization prior to surgery; severe nervous system disease; abnormal mental states; inability to communicate normally.

### Exit criteria

Active request by the patient to withdraw during the study; failure to effectively cooperate with the treatment plan, leading to the failure of normal research protocols; serious accidents during treatment; transfer to an intensive care unit (ICU) for monitoring and treatment after operation.

Before the withdrawal of any subject, the reason for withdrawal was recorded accurately, and a new subject was not included instead.

### Procedures under the research program

Before the surgery, the doctor informed the patient about possible anesthesia methods, possible complications and solutions during anesthesia, and postoperative analgesia strategy. One day before surgery, the patient took laxatives orally to clean the intestines, without undergoing mechanical enema. Solid food was fasted from 6 h before the operation; 2 hours before anesthesia induction, patients took 4 mL/kg of multidimensional carbohydrate drink orally (surgical energy).

After entering the room, patients were monitored for non-invasive blood pressure, electrocardiogram, SpO2, respiratory rate, and body temperature; depth of anesthesia was monitored using a Narcotrend monitor. A FloTrac/Vigileo sensor was connected after radial artery catheterization under local anesthesia to monitor cardiac output (CO), cardiac index (CI), stroke volume (SV), and stroke volume variation (SVV). Before anesthesia induction, midazolam 0.05 mg/kg, pentoxiquine hydrochloride 0.4–0.6 mg, and lansoprazole 30 mg were injected intravenously. Anesthesia induction consisted of etomidate 0.3 mg/kg, sufentanil 0.4–0.6 μ G/kg, cisatracurium 0.15 mg/kg, and mask-assisted respiration, nitrogen removal, and oxygen delivery; the anesthesia machine was connected after endotracheal intubation, with respiratory parameters FiO 30%–50%, tidal volume 6–8 mL/kg, respiratory rate 12–15 times/min, and I:E = 1:2. Subsequently, the right subclavian vein was punctured and catheterized. Ultrasound-guided transabdominal plane (TAP) block was given at the head end of the bilateral iliac crest, with 0.375% ropivacaine 20 mL on each side. Propofol rate was 3–6 mg/(kg/h), remifentanil was 0.05–0.2 μG/(kg/min), and 1%–1.5% sevoflurane was inhaled. During the operation, the concentration of sevoflurane was adjusted according to the Narcotrend value, and the Narcotrend value was maintained at 37–45. Muscle relaxants were added according to the needs of the operation. Ulinastatin 5 000 U/kg was injected into the crystal solution during the operation.

### Fluid management plan

(1) (Daca-Alvarez et al., 2022) Compensatory dilatation (5 mL/kg) was supplemented 30 min before anesthesia induction. Crystalloid solution (Ringer Lactate) was used as the background infusion. Before laparotomy, crystalloid solution (2 mL/kg/h) was input. At the laparotomy stage, 5 mL/(kg/h) was used as the background infusion until the end of the operation (Ding et al., 2022). SVV ≤13% was taken as the reference range of volume reactivity. If SVV >13% at a measured time point, this was defined as a low-volume time point, and volume-loading treatment was carried out: that is, 150 mL of hydroxyethyl starch 130/0.4 sodium chloride injection was given intravenously for 10 min, and SVV was re-evaluated until SVV ≤ 13%. If SVV ≤13% at a measured time point, CI was further evaluated. (Erdem et al., 2022). If CI < 2.5 L/(min/m2), dobutamine 2.5–10 μg/(kg/min) was used. When the heart rate (HR) was higher than 100 beats/min, dobutamine was reduced or stopped. (Pu et al., 2020). Central venous oxygen saturation (ScvO2) was evaluated. When ScvO2 < 73% and Hb < 10 g/dL, concentrated red blood cells were infused to bring Hb ≥ 10 g/dL. If it was still the case that ScvO2<73%, dobutamine 2.5–20 μ G/(kg • min) was used until ScvO2 ≥ 73%.

### Intraoperative management and analgesia plan

When MAP <65 mmHg, intravenous infusion of norepinephrine 40 μg was administered; arterial blood samples were taken every 60 min for blood gas analysis. During the operation, blood glucose was maintained between 5.6 and 10 mmol/L. During the operation, central temperature (nasopharynx temperature) was maintained no lower than 36°C by means of air heater and liquid heating; this was maintained until the patient was transferred back to the ward. Before the end of the operation, 5 mg of dizosine, 6 mg of tropisetron, 5 mg of dexamethasone, 1.5 mg of droperidol, and 0.375% ropivacaine were given for local infiltration anesthesia.

The PCIA formula for postoperative analgesia consisted of deszocin 0.4–0.5 mg/kg, flurbiprofen lipid 5 mg/kg, tropisetron 12–18 mg, background dose 2 mL/h, pressing dose 0.5 mL, locking time 15 min. After the operation, the patient was decannulated and transferred to the PACU. Once the patient was awake, respiration and circulation were stable, and VAS was less than 3 points, he was transferred back to the ward.

### Observed indicators

Mean arterial pressure (MAP), HR, CO, CI, SV, and SVV were recorded after endotracheal intubation (T1), after skin incision (T2), 60 min after pneumoperitoneum (T3), 5 min after laparotomy (T4), at the end of surgery (T5). For this study, the intraoperative fluid infusion volume (crystalloid/colloid), intraoperative blood loss, surgical duration, and amount of vasoactive drugs used during the procedure were recorded. The patient’s time of awakening, status of gas/bowel movements, and postoperative hospital stay were also documented.

### Statistical methods

SPSS 21.0 statistical software was used for statistical analysis. Measurement variables that followed a normal distribution are expressed in the form of x ± s, and repeated-measures ANOVA was used to compare different time points. Differences were regarded as statistically significant if *p* < 0.05.

## Results

### Comparison of patient characteristics

A total of 41 patients who underwent gastrointestinal surgery at our hospital were included in this study, covering a span of 3 years. Table 1 presents demographic data for the patients, including gender, age, and disease type. Statistical analysis revealed no significant differences in these variables among the patient groups (*p* > 0.05).

**TABLE 1:** General information of gastrointestinal surgery.

Group	
Number	41
Age	61.56 ± 10.74
Sex	23 (Man)/18 (Woman)
BMI	23.52 ± 3.52
Cancer category	
Colon cancer	28
Rectal cancer	13
ASA	
ASA-Ⅰ	17
ASA-Ⅱ	24

### Hemodynamic changes during surgery

In comparison to the baseline measurement (T1), there was a significant increase in mean arterial pressure (MAP) from T2 to T5 (*p* < 0.05, Table 2). Cardiac output (CO) was significantly decreased from T2 to T3, but notably increased from T4 to T5 (*p* < 0.05, Table 2). Stroke volume (SV) levels exhibited a significant increase from T3 to T5 (*p* < 0.05, Table 2). Heart rate (HR) and cardiac index (CI) were significantly higher at T1 and T3–T5 than at T2 (*p* < 0.05, Table 1). Moreover, systolic volume variation (SVV) was significantly decreased at T1, T2, T4, and T5 in comparison to T3 (*p* < 0.05, Table 2). At T4 and T5, both CO and SV demonstrated a significant increase (*p* < 0.05, Table 2). These data indicate that GDFT-significant hemodynamic changes were observed during the surgical procedure. GDFT can significantly improve postoperative cardiac function and has potential cardiac protective effects.

**TABLE 2:** Hemodynamic changes during operation.

Group	T1	T2	T3	T4	T5
MAP (mmHg)	80.55 ± 8.31	82.61 ± 7.22*	85.22 ± 10.11*	84.97 ± 7.25*	87.91 ± 6.21*
HR (time/min)	62.32 ± 8.95#	57.74 ± 6.95	60.04 ± 6.68#	66.85 ± 10.05#	61.96 ± 8.52#
CO (L/min)	3.85 ± 0.61	3.22 ± 0.52*	3.84 ± 0.69*	4.33 ± 0.56*^$^	4.07 ± 0.53*^$^
CI [L/(min/m2)]	2.77 ± 0.71#	2.33 ± 0.84	3.03 ± 0.74#	2.71 ± 0.42#	2.75 ± 0.66#
SV (mL)	58.97 ± 9.11	56. 19 ± 8.87	62.53 ± 9.01*	66.05 ± 7.74*^$^	64.18 ± 9.28*^$^
SVV (%)	11.35 ± 2.98^$^	12.98 ± 3.31^$^	13.53 ± 3.37	10.35 ± 2.57^$^	9.42 ± 2.99^$^

*Compared with T1, *p* < 0. 05; # compared with T2, *p* < 0. 05; $compared with T3, *p* < 0. 05.

### Postoperative follow-up

The duration of pressor drug administration was recorded as 23 days in this study. The average time spent in the post-anesthesia care unit (PACU) was 40.22 ± 12.79 min, while the time taken for patients to mobilize and get out of bed was 12.41 ± 3.97 h. The interval prior to bowel movement/defecation was observed to be 18.11 ± 7.52 h. The average length of the postoperative hospital stay was determined to be 4.47 ± 1.98 days (Table 3). GDFT can significantly improve the duration of PACU stay, time to mobilize and get out of bed, and length of hospital stay.

**TABLE 3:** Postoperative follow-up.

Group	
Pressor drug	23
PACU residence time (min)	40.22 ± 12.79
Time for getting out of bed (h)	12.41 ± 3.97
Exhaust and defecation time (h)	18.11 ± 7.52
Postoperative hospital stay (day)	4.47 ± 1.98

### Surgical stress indices

Assessment of surgical stress indices revealed an average HAMA score of 9.11 ± 2.37. Additionally, C-reactive protein (CRP) levels were measured at 10.54 ± 3.38 mg/L, adrenaline levels at 132.87 ± 8.97 ng/L, and cortisol levels at 119.72 ± 4.08 ng/L (Table 4). GDFT can significantly improve surgical stress index measurements.

**TABLE 4:** Surgical stress indexes.

Group	
HAMA (Score)	9.11 ± 2.37
CRP (mg/L)	10.54 ± 3.38
Adrenaline (ng/L)	132.87 ± 8.97
Cortisol (ng/L)	119.72 ± 4.08

### Nutritional status

Regarding patients’ nutritional status, prealbumin levels were found to be 141.98 ± 10.99 mg/L at 3 days after surgery; levels increased to 164.17 ± 15.84 mg/L on the day of discharge from the hospital (Table 5). Lymphocyte counts at 3 days after surgery were reported as 1.22 ± 0.18 (10^9/L), rising to 1.47 ± 0.17 (10^9/L) on the day of discharge (Table 5). Serum albumin levels at 3 days after surgery were recorded as 30.51 ± 2.28 g/L, increasing to 33.52 ± 2.07 g/L on the day of discharge. GDFT can significantly improve postoperative nutritional status.

**TABLE 5:** Nutritional status.

Group	
Prealbumin (mg/L)	
3 d after surgery	141.98 ± 10.99
The day of leave hospital	164.17 ± 15.84
Lymphocyte count (×10^9^ L)	
3 d after surgery	1.22 ± 0.18
The day of leave hospital	1.47 ± 0.17
Serum albumin (g/L)	
3 d after surgery	30.51 ± 2.28
The day of leave hospital	33.52 ± 2.07

## Discussion

Gastrointestinal surgery is characterized by significant trauma, a high risk of blood loss, and a high proportion of elderly patients or those with multiple comorbidities (Harada et al., 2022). The application of Enhanced Recovery After Surgery (ERAS) principles in gastrointestinal surgery has received extensive scholarly attention, and this framework been implemented extensively in clinical practice, gradually reaching a mature stage (Jabłońska et al., 2022). However, fluid management during gastrointestinal surgery is critical, as both fluid overload and inadequate circulating blood volume can have adverse effects on patient outcomes. Specifically, fluid overload can lead to an increased incidence of postoperative complications such as anastomotic leakage and delayed recovery of gastrointestinal function (Pu et al., 2021; Lata et al., 2022). Conversely, insufficient effective circulating blood volume can result in inadequate tissue perfusion, hypoxia, and an elevated risk of postoperative complications and mortality (Mao et al., 2022). In gastrointestinal surgery, devices such as Esophageal Doppler (ED) and FloTrac/Vigileo, among others, are commonly used for real-time monitoring of stroke volume (SV), systolic volume variation (SVV), and pulse pressure variation (PPV) to assess dynamic changes in fluid status and optimize fluid management (Ross et al., 2022). In this study, we collected data from 41 patients who underwent gastrointestinal surgery at our hospital over the past 3 years. Table 1 presents the general characteristics of these patients, including gender, age, and disease type. Makaryus et al. showed that optimization of perioperative fluid management is crucial for Enhanced Recovery Pathways (ERPs), as this helps improve lung function, tissue oxygenation, gastrointestinal motility, and wound healing (Makaryus et al., 2018). Therefore, in this study, data were collected from 41 patients who underwent gastrointestinal surgery to examine the impact of goal-directed fluid therapy on volume management during ERAS in gastrointestinal surgery.

While there has been limited research on the application of goal-directed fluid therapy (GDFT) in gastrointestinal surgery under the ERAS framework, it is worth noting that existing studies have small sample sizes and provide only weak evidence (Scott et al., 2015; Pędziwiatr et al., 2018; Low et al., 2019). Intraoperative GDFT plays a crucial role in perioperative fluid therapy within the ERAS management approach (Sica et al., 2020). Most studies related to ERAS include intraoperative GDFT as a component (Wobith and Weimann, 2021). Therefore, this article aimed to review and discuss the current application and research progress of GDFT in gastrointestinal surgery within the context of ERAS, drawing on high-quality studies. In this study, we observed a significant increase in mean arterial pressure (MAP) from T2 to T5, while cardiac output (CO) was decreased from T2 to T3 but increased from T4 to T5. Grass et al. showed that among these 5,155 patients, 2,320 patients (45.1%) received more than 3 L of intravenous fluids on postoperative day 0 (Grass et al., 2020). Thus, these data also show the encouraging efforts of GDFT, as it has been found to be associated with better outcomes and indicates potential for customization in specific patient populations.

Goal-directed fluid therapy (GDFT) utilizes various observational indicators, including traditional static measurements such as SvO2, central venous pressure, pulmonary capillary wedge pressure, and lactic acid, as well as functional hemodynamic indicators such as stroke volume (SV), SV variation (∆SV), systolic volume variation (SVV), and pulse pressure variation (PPV) (Wobith and Weimann, 2021). Recently, there has been a growing interest in studying SV, ∆SV, SVV, and PPV, and similar parameters measured by equipment such as the FloTrac/Vigileo and LiDCO, as they provide new insights into the cardiac preload of the body (Joosten et al., 2021). It is important to note that SVV or PPV should be evaluated when patients are mechanically ventilated and free from complications such as thoracotomy, arrhythmia, or myocardial disease. In contrast, SV and ∆SV are not influenced by tidal volume or rhythm and can be evaluated in awake patients or those with arrhythmias (Liu et al., 2021). In our study, we observed a significant increase in SV from T3 to T5, while HR and cardiac index (CI) values were notably elevated at T1 and T3–T5. Moreover, CO and SV demonstrated a significant increase at T4 and T5. Makaryus et al. showed that GDFT improves blood flow intraoperatively, and ultimately reduce LOS and complications (Makaryus et al., 2018). Therefore, this treatment method improves pulmonary function, tissue oxygenation, gastrointestinal motility, and wound healing. The clinical demand for evidence-based Enhanced Recovery After Surgery (ERAS) programs is increasing with the rise in the number of patients undergoing major surgeries with medium to high risk levels (McLain et al., 2021). Early research on ERAS has shown that optimizing intraoperative fluid management can significantly benefit patients (Mizunoya et al., 2019). The debate surrounding intraoperative fluid management schemes has evolved from traditional rehydration approaches to restrictive rehydration and ultimately to individualized goal-directed fluid therapy (GDFT) supported by evidence-based medicine (Mohammed El-Hadi Shoukat Mohammed et al., 2021). Numerous high-quality studies have demonstrated the significant advantages of GDFT for medium-to high-risk patients undergoing major surgery, including a reduction in the incidence and mortality of postoperative complications, shorter hospital stays, lower medical costs, faster postoperative recovery, and improved quality of life, aligning with the principles of ERAS (Rollins et al., 2020). It is worth noting that perioperative fluid management under the ERAS model should encompass the entire diagnostic and treatment process, including the stages before, during, and after surgery, as fluid management at each stage can significantly impact patient prognosis (Turi et al., 2021). In this study, the duration of pressor drug administration was recorded as 23 days, while the average time spent in the post-anesthesia care unit (PACU) was 40.22 ± 12.79 min. Patients took an average of 12.41 ± 3.97 h to mobilize and get out of bed, and 18.11 ± 7.52 h for bowel movements/defecation; the average length of the postoperative hospital stay was 4.47 ± 1.98 days. Additionally, the average HAMA score was determined to be 9.11 ± 2.37; CRP levels were measured at 10.54 ± 3.38 mg/L, adrenaline levels at 132.87 ± 8.97 ng/L, and cortisol levels at 119.72 ± 4.08 ng/L. Lee et al. reported that goal-directed fluid therapy is associated with earlier progression to a postoperative soft diet, reduced acute postoperative pain intensity, and less rescue analgesics (Lee et al., 2021). Thus, these data also illustrate the fact that goal-directed fluid therapy leads to better postoperative recovery and a better renal protective effect.

However, it is important to acknowledge that inappropriate treatment at any stage of the diagnostic and treatment process can undermine the entire ERAS pathway and affect the final outcome (Turi et al., 2022). This article has primarily focused on intraoperative fluid management within the ERAS framework, without delving into preoperative and postoperative fluid management schemes (Virág et al., 2022). Due to the current medical model and the roles assigned in implementing the overall ERAS program, anesthesiologists are primarily responsible for controlling and accurately managing the intraoperative stages of fluid management, while preoperative and postoperative fluid management is often carried out by surgeons (Xie et al., 2021). This approach contradicts the concept of continuous and unified fluid management advocated by anesthesiologists during the perioperative period (Weinberg et al., 2019; Zorrilla-Vaca et al., 2021). Regarding nutritional status, in this study, prealbumin levels were found to be 141.98 ± 10.99 mg/L at 3 days after surgery, increasing to 164.17 ± 15.84 mg/L upon discharge from the hospital. Lymphocyte counts at 3 days after surgery were reported to be 1.22 ± 0.18 (10^9/L), increasing to 1.47 ± 0.17 (10^9/L) on the day of discharge. Serum albumin levels at 3 days after surgery were recorded as 30.51 ± 2.28 g/L, increasing to 33.52 ± 2.07 g/L on the day of discharge. These data indicate that goal-directed fluid therapy has a better effect at discharge.

While the concept of perioperative fluid management has been established for some time, its implementation in clinical practice lacks uniformity within the current medical development model. However, with the emergence of perioperative medicine as a specialized discipline, it is expected that a standardized approach to managing the perioperative process will be developed in the future. This may enable anesthesiologists to implement continuous and consistent fluid management for patients undergoing elective surgery.

It is worth mentioning that this article has specifically focused on the application of GDFT in gastrointestinal surgeries under the ERAS model, without covering its utilization in other areas. Additionally, this article has not delved extensively into preoperative and postoperative fluid management schemes and their impact on patients. Finally, due to the limited sample size of 41 cases from the author’s institution, further validation through larger-scale studies is required to confirm the findings of this study.

## Data Availability

The raw data supporting the conclusion of this article will be made available by the authors, without undue reservation.

## Abbreviations

ERAS, enhanced recovery after surgery; TAP, transabdominal plane; GDFT, goal-directed fluid therapy; CO, cardiac output; CI, cardiac index; SV, stroke volume; SVV, stroke volume variation; MAP, mean arterial pressure; HR, heart rate.
